# Palliative Transcatheter Arterial Embolization for Life-Threatening Hemorrhage from Advanced Buccal Mucosal Carcinoma: A Case Report

**DOI:** 10.3390/reports9040340

**Published:** 2026-10-08

**Authors:** Yoshiyuki Miyamoto, Keigo Kubota, Hiroshi Kawakami, Atsushi Hosono, Yasuhiko Tsuyama

**Affiliations:** 1 Division of Dentistry and Oral Surgery, Mitsui Memorial Hospital, Tokyo 101-8643, Japan; miyamotoy-ora@h.u-tokyo.ac.jp (Y.M.); kakashi554@gmail.com (H.K.); godhand1996@gmail.com (Y.T.); 2 Department of Oral-Maxillofacial Surgery and Orthodontics, The University of Tokyo Hospital, Tokyo 113-8655, Japan; 3 Division of Neurosurgery, Mitsui Memorial Hospital, Tokyo 101-8643, Japan; ratopani.hot.spring@gmail.com

**Keywords:** buccal mucosal carcinoma, transcatheter arterial embolization, tumor hemorrhage, n-butyl-2-cyanoacrylate, case report

## Abstract

**Background and Clinical Significance:** Hemorrhage from advanced oral cancer can threaten both circulation and airway patency. **Case Presentation:** A 60-year-old man with advanced left buccal mucosal squamous cell carcinoma (cT4bN2bM0, Stage IVB) was medically unsuitable for radical surgery. Definitive radiotherapy was initiated, but after 20 Gy in 10 fractions, massive tumor hemorrhage on 12 July 2022 posed an imminent risk of airway obstruction and required intubation. After local hemostatic measures and transfusion, transcatheter arterial embolization (TAE) of tumor-feeding external carotid branches was performed on 14 July using coils and n-butyl-2-cyanoacrylate. Angiography showed markedly reduced tumor staining; the patient was extubated the next day. No recurrent tumor bleeding or clinically apparent neurological deficit was documented through the last outpatient evaluation on 8 August 2022. **Conclusions:** TAE achieved short-term hemostasis and facilitated early extubation in this patient; durable efficacy and survival benefit cannot be established from this case.

## 1. Introduction and Clinical Significance

Hemorrhage is a potentially catastrophic complication of advanced head and neck cancer. Tumor necrosis, infection, treatment-related tissue injury, and vascular invasion can cause recurrent or massive bleeding, resulting in hemorrhagic shock, aspiration, and acute airway obstruction. Conventional measures such as compression, electrocautery, suturing, and surgical ligation may be ineffective when tumor tissue is friable or when bleeding arises from multiple deep vessels [1].

Endovascular embolization permits selective or superselective occlusion of culprit arteries and has been used for hemorrhage related to trauma, vascular malformations, postoperative complications, and advanced malignancy. Published series of patients with advanced head and neck cancer suggest that embolization can achieve rapid hemostasis in a substantial proportion of patients treated with palliative intent [2,3,4,5,6]. However, the procedure carries risks including non-target embolization and neurological complications, and treatment should be individualized according to vascular anatomy, clinical status, and goals of care.

We describe emergency TAE for hemorrhage from advanced buccal mucosal squamous cell carcinoma in a patient medically unsuitable for radical surgery. This report focuses on the procedural approach, short-term hemostasis, airway management, and documented follow-up, in accordance with CARE reporting principles.

In contrast to broader series of embolization for head and neck tumor hemorrhage, this report documents the sequence of airway-protective intubation, multibranch external carotid embolization, and next-day extubation in a patient with advanced buccal mucosal carcinoma, together with the limits of short-term follow-up. This clinical sequence, rather than a previously unreported embolization technique or a claim of priority, is the specific contribution of the present report.

## 2. Case Presentation

A 60-year-old man presented on 27 May 2022 with left cheek swelling. His medical history included pancreatogenic diabetes (HbA1c, 9.4%), hypertension, liver abscess, portal vein thrombosis, previous myocardial infarction, chronic pancreatitis, and nephrotic syndrome associated with diabetic nephropathy. He was 163 cm tall and weighed 53 kg. He had smoked 20 cigarettes daily for 30 years and consumed three 500-mL bottles of beer daily.

**Figure 1:**
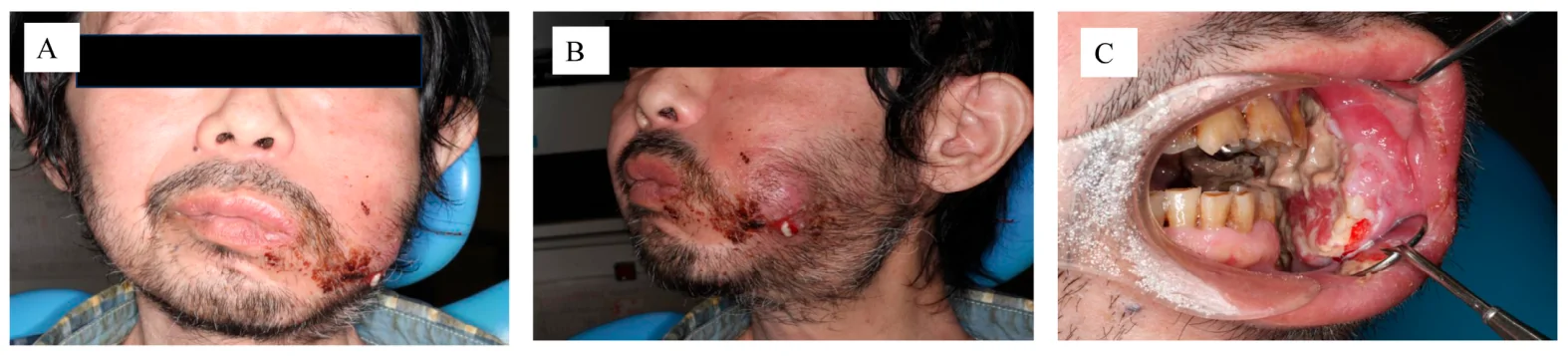
Clinical findings at initial presentation. (**A**,**B**) Erythema, swelling, and induration of the left cheek. (**C**) Ulcerative lesion with surrounding induration of the left buccal mucosa.

Extraoral examination showed facial asymmetry with erythema, swelling, and induration of the left cheek, accompanied by restricted mouth opening (Figure 1A,B). Intraoral examination demonstrated an ulcerative lesion with surrounding induration of the left buccal mucosa, measuring approximately 80 × 40 × 30 mm (Figure 1C).

**Figure 2:**
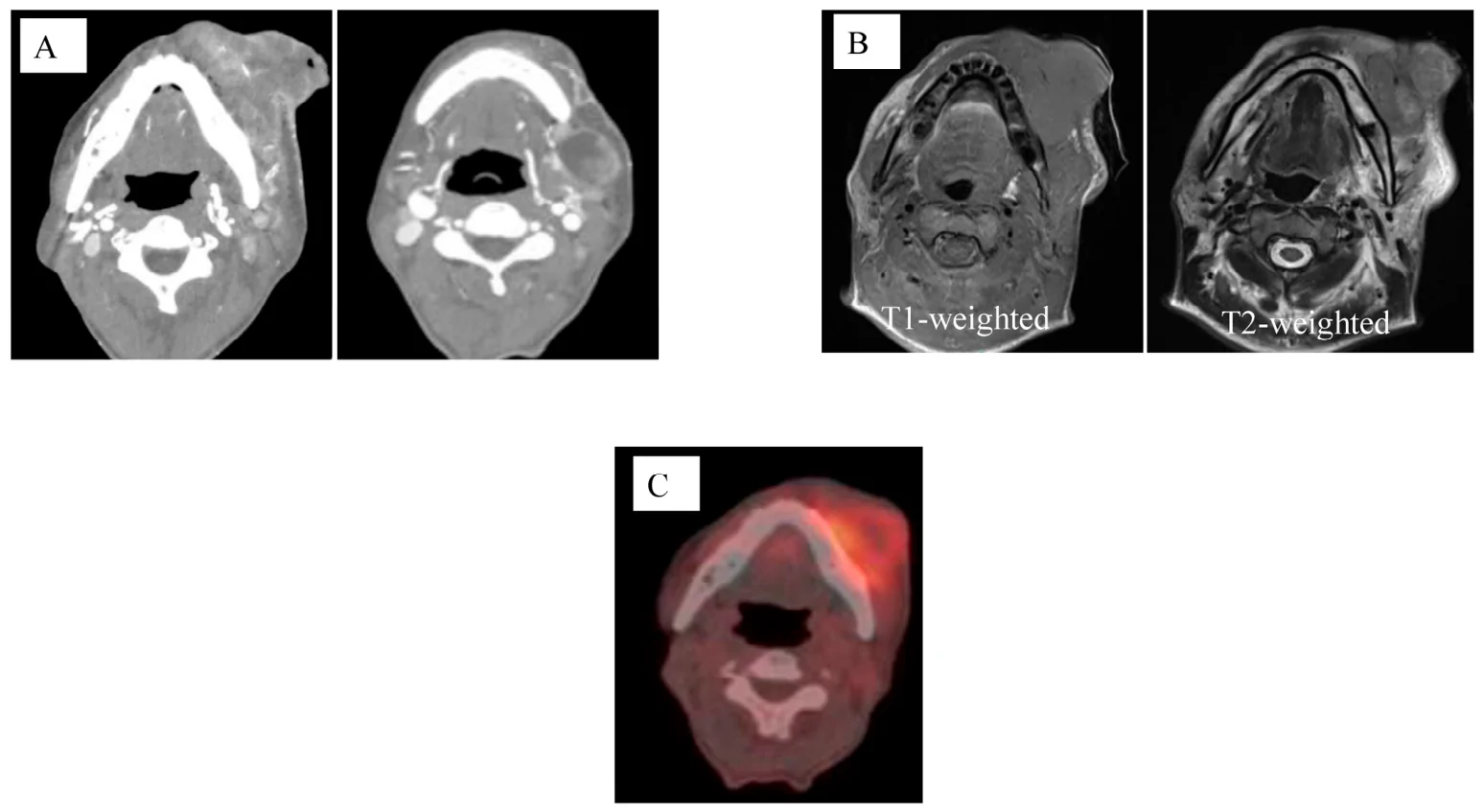
Imaging findings at diagnosis. (**A**) Contrast-enhanced CT showing the left buccal mucosal tumor and enlarged left cervical lymph nodes. (**B**) MRI showing deep tumor invasion with suspected extension to the lateral pterygoid muscle. (**C**) FDG-PET/CT showing increased uptake in the primary lesion (SUVmax 7.4).

Contrast-enhanced CT showed a left buccal mucosal mass and enlarged ipsilateral cervical lymph nodes (Figure 2A). Contrast-enhanced CT obtained on 20 June 2022 demonstrated tumor invasion into the masticator space (Supplementary Figure S1), supporting cT4b. MRI obtained on 15 June 2022 showed multiple ipsilateral submandibular lymph nodes (Supplementary Figure S2). Three ipsilateral submandibular lymph nodes considered metastatic on imaging measured 8.3, 27.7, and 6.5 mm. The 27.7-mm and 6.5-mm lymph nodes demonstrated central necrosis, while the anterior 8.3-mm lymph node showed a mildly ill-defined posterior margin and heterogeneous internal appearance. None of these lymph nodes demonstrated imaging findings sufficient to establish definite extranodal extension (ENE). These findings supported a clinical nodal classification of cN2b. Clinical staging was cT4bN2bM0, Stage IVB, according to the UICC TNM Classification of Malignant Tumours, eighth edition. FDG-PET/CT performed for staging showed no evidence of distant metastasis, supporting cM0 (Figure 2C). The patient was admitted on 1 June 2022. Cytology showed malignant cells (Papanicolaou class V); biopsy on 6 June confirmed squamous cell carcinoma on 13 June 2022, reported as histological grade 1 according to the WHO grading system and Yamamoto–Kohama invasion pattern YK-3. YK-3 is characterized by groups of tumor cells with no distinct borderline at the invasive front [7].

**Figure 3:**
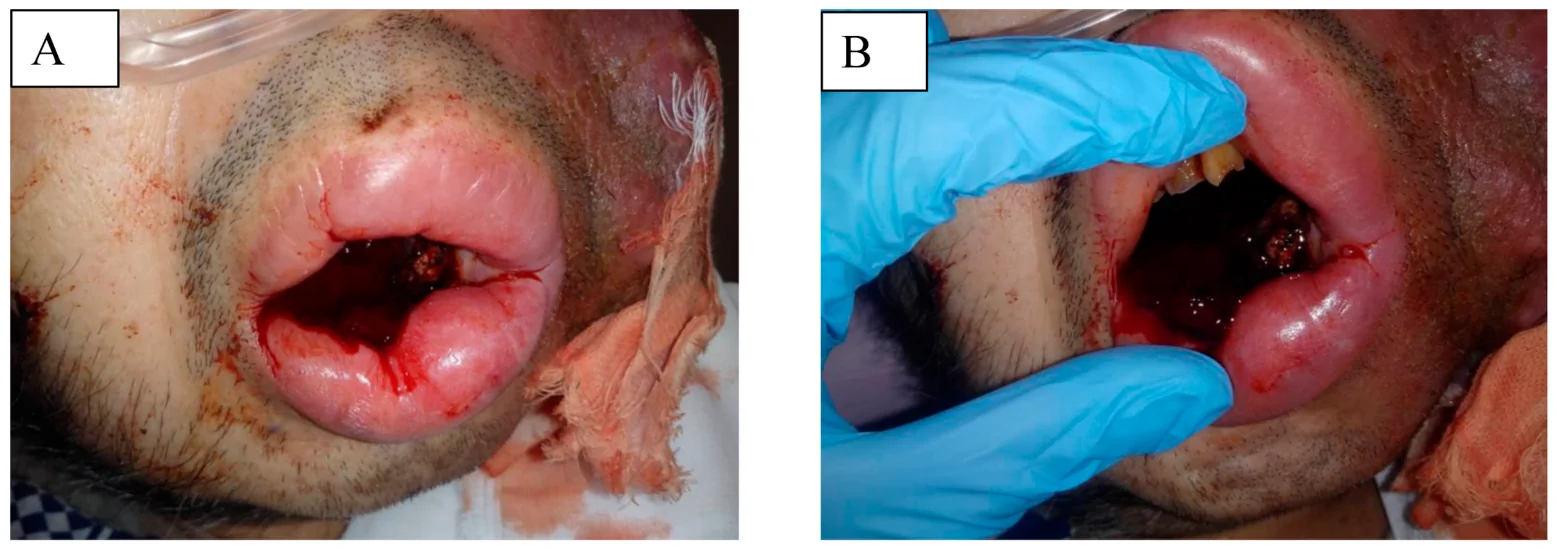
Oral findings during the hemorrhagic episode on 12 July 2022 before embolization. (**A**,**B**) Tumor-associated oral bleeding and swelling posed an imminent risk of airway obstruction.

Tumor resection with immediate reconstruction and left neck dissection was initially considered. Following multidisciplinary assessment, the patient was judged medically unsuitable for radical surgery because of systemic comorbidities. He was discharged on 24 June 2022. Definitive radiotherapy was started on 28 June 2022, planned as 70 Gy in 35 fractions (2 Gy per fraction). Ten fractions (20 Gy) were delivered on 28, 29, and 30 June and on 1, 4, 5, 6, 7, 8, and 11 July 2022. On 12 July 2022, oral tumor hemorrhage (estimated blood loss, 400–500 mL) posed an imminent risk of airway obstruction and necessitated endotracheal intubation for airway protection (Figure 3A,B). Hemoglobin was 8.5 g/dL and platelets 20.1 × 10^4^/µL on 20 June, compared with 7.8 g/dL and 21.2 × 10^4^/µL on 12 July. Because of his history of portal vein thrombosis, the patient had been receiving warfarin 1.25 mg once daily before hospitalization. Because mild tumor-related bleeding had persisted from the initial presentation, warfarin was discontinued on 7 July 2022 after consultation with the treating physician; no anticoagulation reversal agent was administered. Prolongation of PT-INR and aPTT was observed during the clinical course, including before the major hemorrhagic event and TAE. The available medical records did not document a definitive explanation for these abnormalities other than the possible contribution of anticoagulant therapy. The serial platelet counts and coagulation parameters are shown in Supplementary Figure S3. Blood pooled in the oral cavity was removed as far as possible; oxidized regenerated cellulose (Surgicel; Ethicon, Inc., Raritan, NJ, USA) was applied with compression, and carbazochrome sodium sulfonate (Adona; Mitsubishi Tanabe Pharma Corporation, Osaka, Japan) and tranexamic acid were administered. On 12 July, the patient received four Japanese units of red-cell concentrate (560 mL) and 12 Japanese units of fresh frozen plasma (720 mL); two additional Japanese units of red-cell concentrate (280 mL) were administered on 13 July. Radiotherapy was interrupted after the hemorrhage. Because safe extubation was unlikely without reliable hemostasis, the neurosurgical team was consulted, informed consent was obtained, and TAE was performed on 14 July 2022.

Under general anesthesia with nasotracheal intubation, the patient underwent TAE in a hybrid operating room (procedure time, 93 min). A 6-Fr SuperSheath (25 cm; Medikit Co., Ltd., Tokyo, Japan) was placed via the right femoral artery. Using a 4-Fr JB2 angiographic catheter (125 cm; Medikit Co., Ltd., Tokyo, Japan) and 0.035-inch Radifocus guidewire (180 cm; Terumo, Tokyo, Japan), left internal and external carotid angiography was performed. No tumor staining was seen in the internal carotid circulation. External carotid angiography demonstrated tumor supply from the facial, submental, maxillary (including buccal and descending palatine), superficial temporal (including transverse facial), and zygomatico-orbital arterial branches. Potential external-to-internal carotid and ophthalmic collateral pathways were assessed by selective angiography, particularly near the angular and middle meningeal arterial territories. A 6-Fr Optimo guiding catheter (100 cm, STR; Tokai Medical Products, Kasugai, Japan) was placed in the left external carotid artery for temporary flow control.

**Figure 4:**
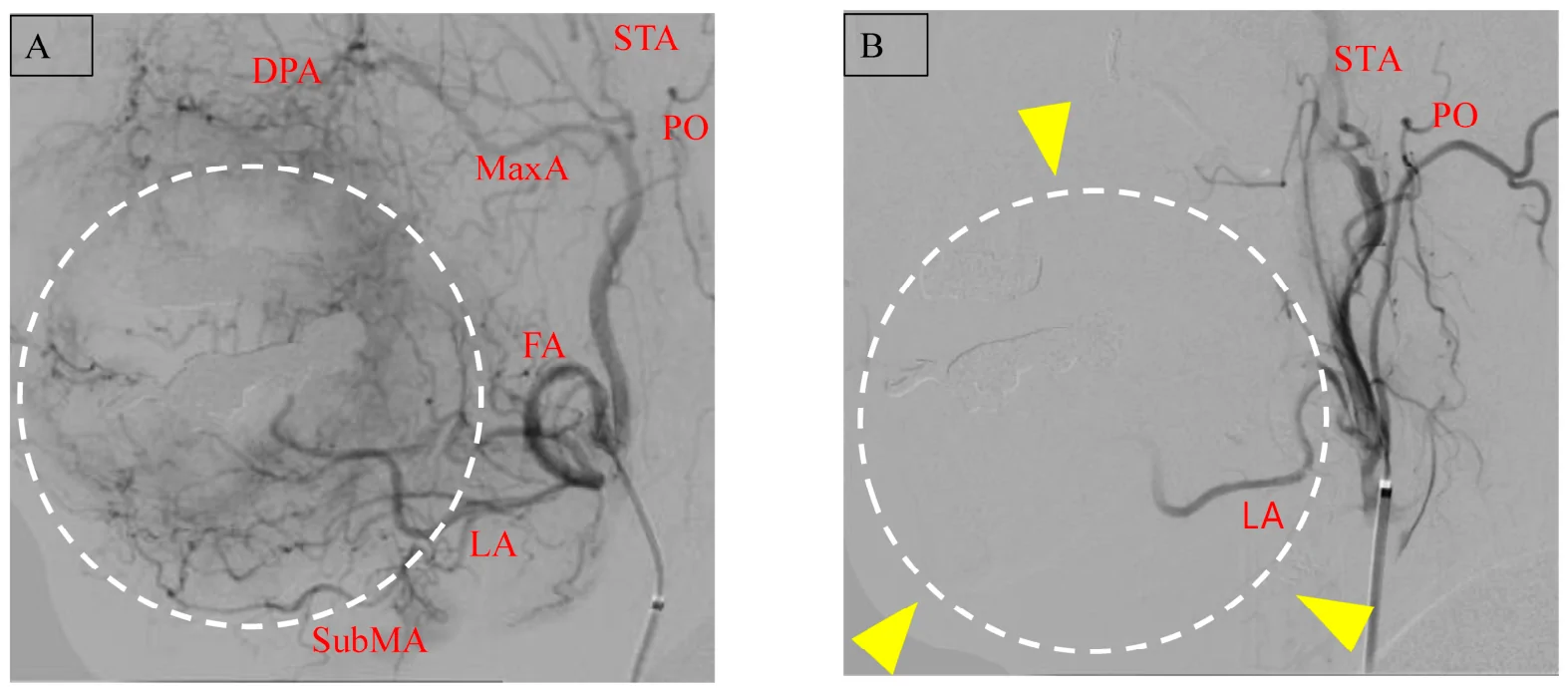
External carotid angiography before (**A**) and after (**B**) embolization. Dashed circles delineate the tumor-staining region; yellow arrowheads indicate markedly attenuated tumor staining after embolization. LA, lingual artery, which was assessed angiographically but was not embolized. Abbreviations: ICA, internal carotid artery; ECA, external carotid artery; FA, facial artery; SubMA, submental artery; MaxA, maxillary artery; STA, superficial temporal artery; LA, lingual artery; BA, buccal artery; DPA, descending palatine artery; TFA, transverse facial artery; ZOA, zygomatico-orbital artery.

An Excelsior SL-10 microcatheter (straight, two-tip marker, 150 cm × 6 cm; Stryker Neurovascular, Fremont, CA, USA) with a 0.014-inch ASAHI CHIKAI microwire (200 cm; Asahi Intecc Co., Ltd., Seto, Japan) was advanced into the facial artery. Because distal access was limited and flow was high, an Axium Prime Helix coil (2.5 mm × 8 cm; Medtronic, Minneapolis, MN, USA) was placed proximally; following a 5% dextrose flush, 0.13 mL of a 20% n-butyl-2-cyanoacrylate (NBCA)–Lipiodol mixture (NBCA:Lipiodol = 1:4) was injected under temporary external carotid flow control. Selective lingual angiography showed no substantial tumor staining, and the lingual artery was preserved. The submental artery was embolized with 0.14 mL of the same 20% NBCA–Lipiodol mixture after a dextrose flush. The zygomatico-orbital and transverse facial arterial supply was treated via the superficial temporal artery using 0.16 mL of the same mixture. Finally, a Headway 17 microcatheter (1.7/2.4 Fr, 150 cm, straight, two markers; Terumo Clinical Supply, Gifu, Japan) was positioned near the proximal buccal artery; an Axium Prime Helix coil (3 mm × 10 cm; Medtronic, Minneapolis, MN, USA) was placed in the distal third segment of the maxillary artery to limit distal migration, and 0.14 mL of the 20% NBCA–Lipiodol mixture was injected to embolize the buccal and descending palatine arterial supply. Final angiography demonstrated marked attenuation of tumor staining without internal carotid occlusion or slow flow (Figure 4A,B). Femoral access was closed using Perclose ProGlide (Abbott Vascular, Santa Clara, CA, USA).

**Figure 5:**
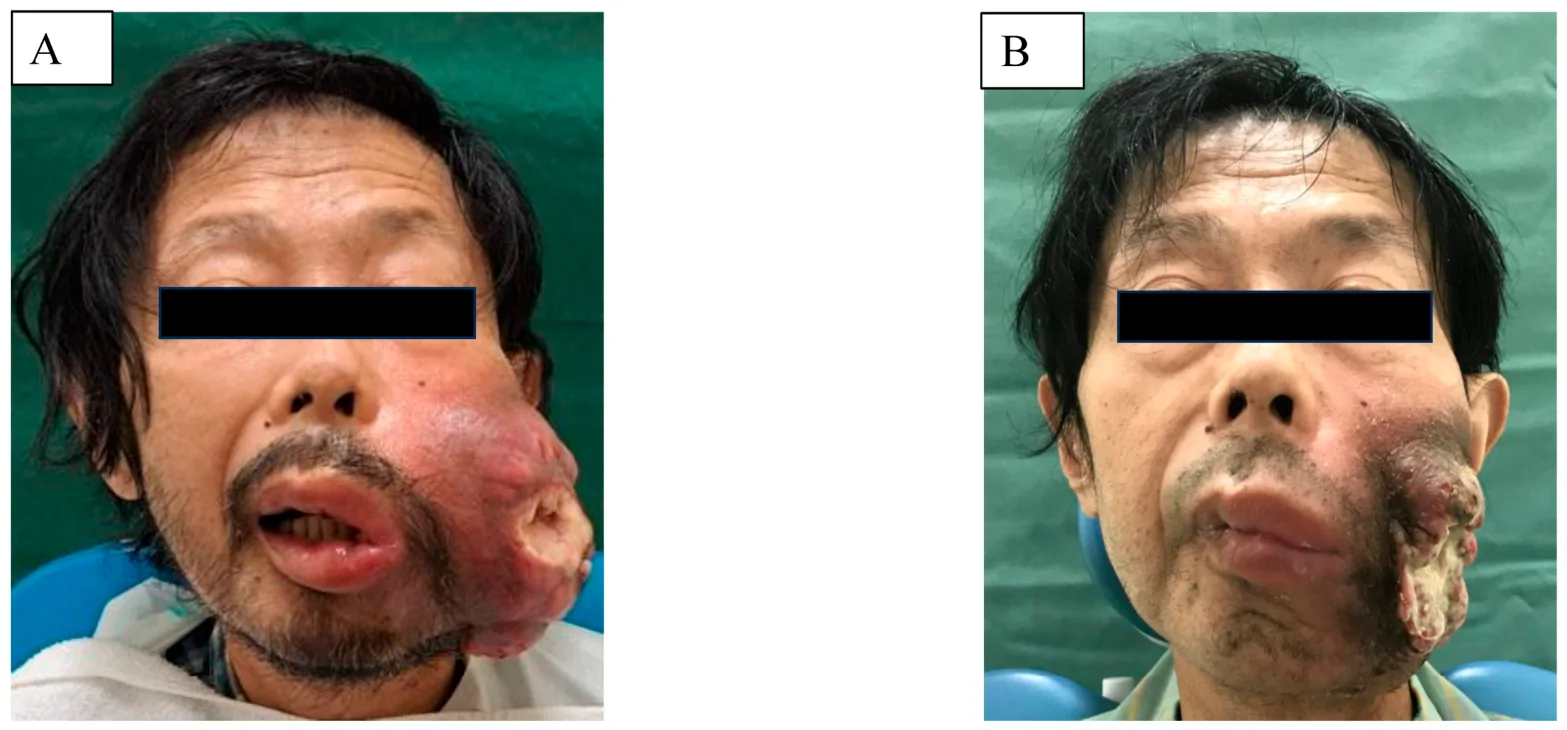
Facial findings before and after TAE. (**A**) On 28 June 2022, the day radiotherapy was initiated, before embolization. (**B**) Outpatient follow-up on 28 July 2022, 14 days after TAE.

**Figure 6:**
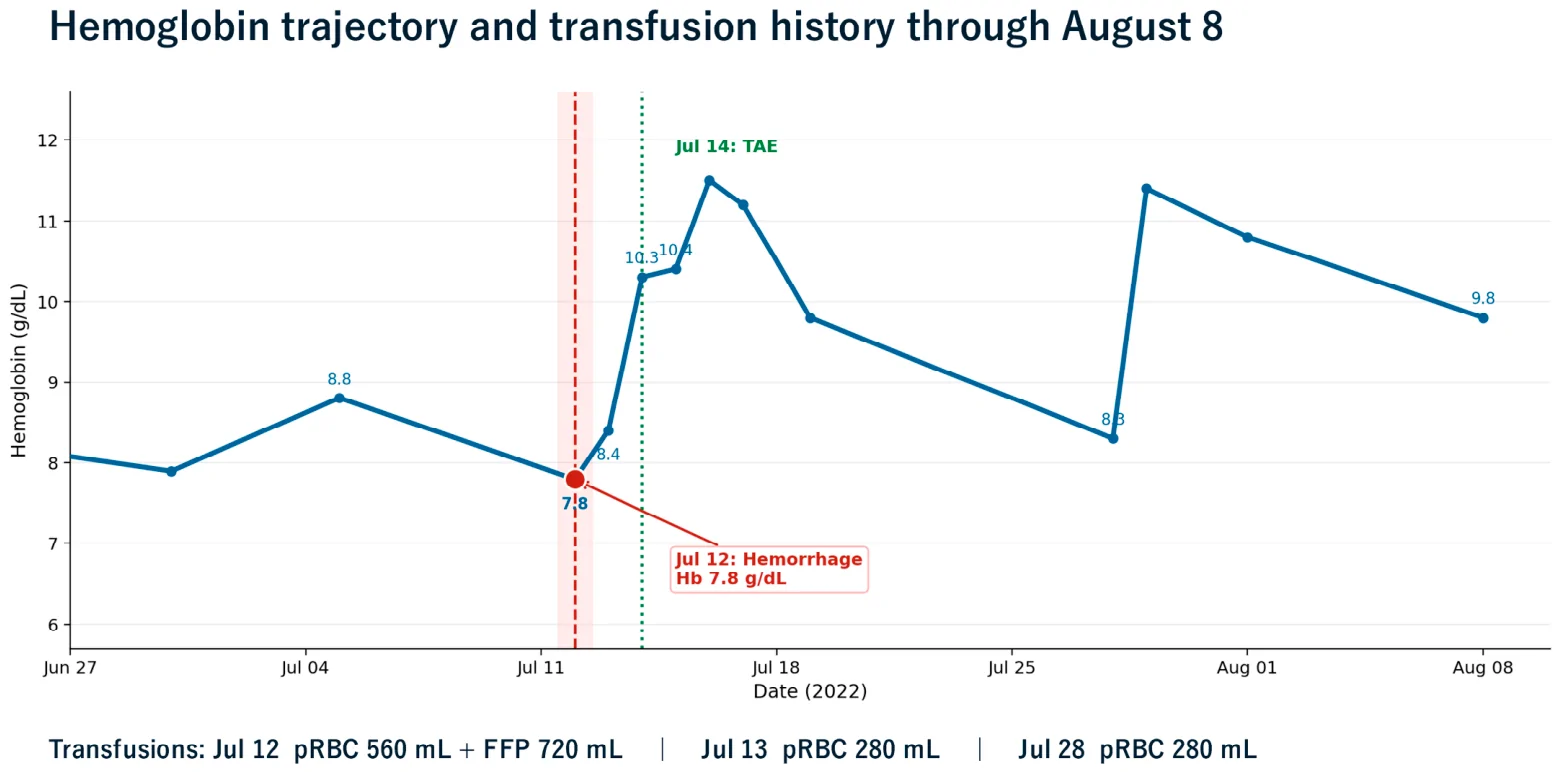
Hemoglobin trajectory and transfusion history through 8 August 2022. The major tumor hemorrhage occurred on 12 July and TAE was performed on 14 July. Red-cell concentrate and fresh frozen plasma were administered on 12 July, followed by red-cell concentrate on 13 July. Red-cell concentrate (280 mL) was also administered on 28 July for anemia, without recurrent tumor hemorrhage.

After TAE, no further tumor hemorrhage was documented during admission. Clinically apparent intraoral and external cheek swelling decreased, and the patient was extubated on 15 July 2022. Oral intake resumed, and he was discharged on 25 July after consent had been obtained for emergency tracheostomy if recurrent bleeding threatened the airway. Facial findings before and after TAE are shown in Figure 5. At the outpatient visit on 28 July, a packed red blood cell transfusion (280 mL) was administered for anemia, not recurrent hemorrhage; no oral bleeding, airway compromise, oral-intake difficulty, or clinically apparent neurological deficit, including facial weakness or symptoms of stroke, was observed. Contrast-enhanced head and neck CT was obtained on 6 August to assess the lesion; quantitative tumor-volume change was not established. The hemoglobin trajectory and transfusion history are summarized in Figure 6. At the last documented outpatient visit on 8 August, no recurrent bleeding or clinically apparent neurological abnormality was observed; hemoglobin was 9.8 g/dL and platelets 17.6 × 10^4^/µL. Radiotherapy resumption was considered after hemostasis, but his general condition deteriorated in early September and treatment was not resumed. He died of heart failure on 6 September 2022; no autopsy was performed.

**Figure 7:**
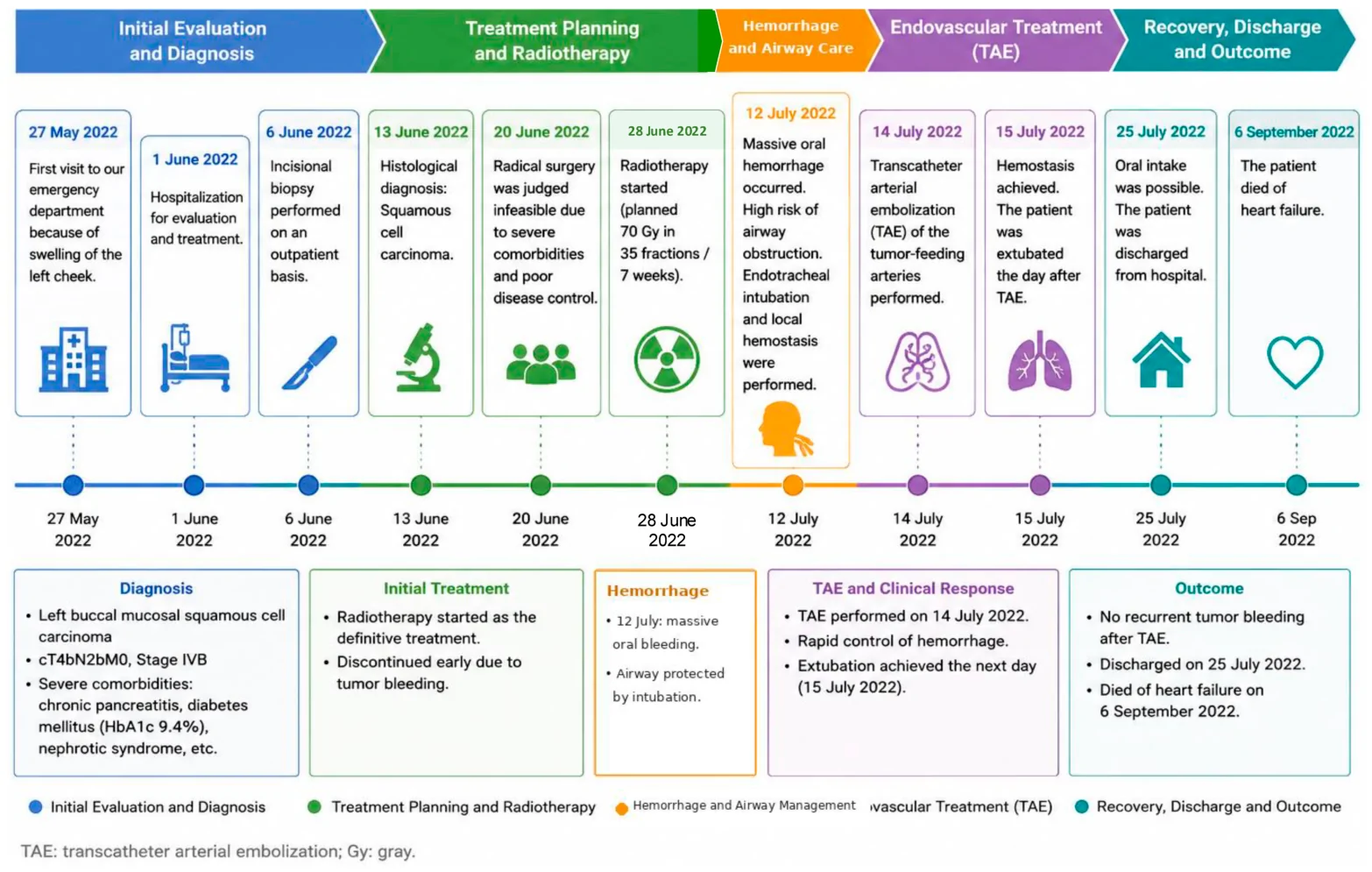
Clinical timeline. Definitive radiotherapy was delivered on 28, 29, and 30 June and on 1, 4, 5, 6, 7, 8, and 11 July 2022 (20 Gy in 10 fractions); major hemorrhage and intubation occurred on 12 July; TAE on 14 July; extubation on 15 July; discharge on 25 July; outpatient review and anemia-related transfusion on 28 July; follow-up CT on 6 August; last documented outpatient assessment on 8 August; and death from heart failure on 6 September 2022.

The overall clinical course, including radiotherapy, the hemorrhagic event, TAE, and subsequent follow-up, is summarized in Figure 7.

## 3. Discussion

This case illustrates short-term control of hemorrhage from an advanced buccal mucosal carcinoma following TAE of multiple external carotid arterial branches, with extubation on the following day. The observed benefit was local hemostasis during the documented follow-up period; a causal effect on survival cannot be inferred.

The contribution of this case is its detailed documentation of the airway-threatening presentation and subsequent airway course following multibranch embolization; it does not establish that this combination is unique or that embolization caused the observed reduction in swelling.

Published studies of endovascular treatment for malignant oral cavity and head and neck hemorrhage provide the most relevant context for this case (Table 1). Kakizawa et al. treated oral hemorrhage in 10 patients with malignant head and neck tumors using 13 emergency embolization procedures; technical success was achieved in 13/13 procedures and overall clinical success was 92%, while recurrent bleeding occurred in 2/9 procedures with available one-month follow-up [2]. In a palliative series of 76 patients with extensive head and neck tumors, Rzewnicki et al. reported cessation of hemorrhage in 65 patients (86%); four patients experienced recurrent hemorrhage within hours requiring other treatment, and seven had less severe recurrence after approximately two days and underwent repeat embolization [6].

Chatani et al. studied 31 patients undergoing 40 emergency TAE procedures for head and neck cancer-associated hemorrhage. Technical success was 100%, while rebleeding was reported in 17.2% of patients at 30 days and 35.5% over follow-up [5]. This cohort included 10 patients with carotid blowout syndrome (CBS), a distinct hemorrhagic mechanism from the tumor-associated external-carotid-branch bleeding in our patient; three patients with CBS experienced cerebral infarction [5]. In a more directly comparable oral cavity cohort, Dang Nguyen Van et al. reported initial hemostasis in 16/16 patients who experienced acute hemorrhage during concurrent chemoradiotherapy, with one patient experiencing recurrent bleeding three weeks later [8]. Our patient received radiotherapy without concurrent chemotherapy. These studies differ in tumor sites, bleeding mechanisms, denominators, definitions of success, and follow-up; their reported rates should not be compared as equivalent estimates.

The present patient underwent embolization of multiple external carotid artery branches after severe tumor-associated bleeding on 12 July 2022. Hemostasis was observed after TAE, extubation was achieved the following day, and no recurrent tumor hemorrhage was documented during follow-up through 8 August 2022. The red-cell transfusion on 28 July was administered for anemia, not recurrent bleeding. These observations establish short-term clinical control in this patient but do not demonstrate durable prevention of hemorrhage or a survival benefit.

Other retrospective series have examined embolization in patients with advanced head and neck cancer [3,4,9]. Their observations provide additional clinical context, but differences in selection and treatment prevent causal conclusions about survival or quality of life. CBS involves rupture of the carotid arterial system and should not be equated with tumor-surface hemorrhage from external carotid branches [10,11].

Coils and liquid embolic agents can be used to occlude tumor-feeding vessels, but their deployment requires careful angiographic assessment of external-to-internal carotid and ophthalmic arterial anastomoses to reduce the risk of non-target embolization and neurological injury [2,5,12,13,14]. In this patient, coils were used for flow control before delivery of n-butyl-2-cyanoacrylate (NBCA). Multidisciplinary planning addressed hemorrhage control and airway protection. This case does not establish an indication for prophylactic embolization in the absence of active bleeding.

Whether earlier angiographic assessment would have altered the clinical course in this patient is unknown. The decision to perform angiographic assessment and embolization must balance the anticipated hemostatic benefit against non-target embolization and tissue-ischemia risks, taking account of the patient’s clinical condition and goals of care.

Potential complications of embolization include ischemic injury to cranial nerves supplied by external carotid branches, mucosal or cutaneous necrosis from excessive or non-target tissue devascularization, and stroke or visual injury if embolic material passes through external-to-internal carotid or ophthalmic arterial anastomoses [15]. In the present case, internal and external carotid angiography and selective assessment of collateral pathways, particularly near the angular and middle meningeal territories, informed vessel selection. Coils and temporary flow control were used before NBCA delivery, and the lingual artery was preserved because it did not show substantial tumor staining. No clinically apparent neurological deficit was documented during follow-up; systematic neurological imaging or standardized assessment for delayed mucosal necrosis was not reported.

Limitations include the single-patient design, lack of quantitative tumor-volume assessment, and bleeding follow-up documented only through 8 August 2022. The patient subsequently died of heart failure, limiting longer-term assessment. The estimated blood loss and clinically apparent changes in swelling were not standardized measurements.

The four reported injections of 20% NBCA–Lipiodol mixture (NBCA:Lipiodol = 1:4) totaled 0.57 mL (0.13, 0.14, 0.16, and 0.14 mL); the individual target vessels, catheter systems, coil dimensions, and angiographic precautions are described in the Case Presentation. No recurrent tumor hemorrhage was documented between TAE on 14 July and the last outpatient assessment on 8 August 2022 (25 days). Although extubation was achieved on 15 July and clinically apparent cheek swelling decreased, standardized pre- and postprocedural swelling measurements, ventilator settings, oxygenation indices, and formal extubation-readiness parameters were not available; these observations should not be interpreted as quantified tumor regression or proof of a causal airway benefit.

## 4. Conclusions

TAE of tumor-feeding external carotid branches was followed by cessation of documented hemorrhage and successful extubation the following day in a patient with advanced buccal mucosal carcinoma. No recurrent bleeding was documented through the last outpatient assessment on 8 August 2022. This case supports consideration of TAE as a local hemostatic option in selected patients when conventional measures are insufficient, without establishing long-term efficacy or survival benefit.

### Patient Perspective

A patient-authored perspective on the treatment was not available because the patient died before preparation of this case report.

## Figures and Tables

**Table 1:** Published studies of transcatheter arterial embolization (TAE) for hemorrhage associated with oral cavity and head and neck malignancies.

Study	Patients	Hemostatic Outcomes	Rebleeding
Dang Nguyen Van et al., 2024 [8]	16	16/16	1/16
Kakizawa et al., 2005 [2]	10	92% overall clinical success	2/9 procedures (22%; one-month follow-up)
Rzewnicki et al., 2012 [6]	76	65/76 (86%)	11/76 (14.5%): 4 within hours; 7 approximately 2 days later, requiring repeat embolization.
Chatani et al., 2021 [5]	31	Technical success: 40/40 procedures (100%)	17.2% at 30 days

Note: Hemostatic success and rebleeding outcomes have different denominators and follow-up periods across studies; direct comparisons are not appropriate. Abbreviations: TAE, transcatheter arterial embolization. Hemostatic success and rebleeding outcomes were reported using different denominators and follow-up periods across studies. The reported technical success rates are procedure-based, whereas other outcomes may be patient-based. Therefore, direct comparisons of outcomes between studies should be interpreted with caution.

## Data Availability

The data presented in this case report are available from the corresponding author on reasonable request, subject to institutional and patient-privacy restrictions.

## Supplementary Materials

The following supporting information can be downloaded at: https://www.mdpi.com/article/10.3390/reports9040340/s1. Supplementary Figure S1. Contrast-enhanced computed tomography obtained on 20 June 2022. The tumor invades the masticator space (arrowhead). Supplementary Figure S2. Magnetic resonance imaging obtained on 15 June 2022. Three ipsilateral submandibular lymph nodes considered metastatic on imaging are indicated (arrowheads). The lymph nodes measured 27.7, 8.3, and 6.5 mm. The 27.7-mm and 6.5-mm lymph nodes showed central necrosis; the anterior 8.3-mm lymph node showed a mildly ill-defined posterior margin and heterogeneous internal appearance. Definite extranodal extension was not established on imaging. Supplementary Figure S3. Serial laboratory parameters before and after tumor hemorrhage. Serial platelet counts and coagulation parameters (PT-INR and aPTT) are shown. The institutional reference ranges were PT-INR 0.85–1.15 and aPTT 20.0–40.0 s. The hemorrhagic event on 12 July 2022 and transcatheter arterial embolization on 14 July 2022 are indicated. Hemoglobin and transfusion history are shown separately in Figure 6.
