# Supplementary material for: Palliative Transcatheter Arterial Embolization for Life-Threatening Hemorrhage from Advanced Buccal Mucosal Carcinoma: A Case Report
Source: Reports (MDPI). 2026 Oct 8;9(4):340. doi: 10.3390/reports9040340 (PMC13692386; doi:10.3390/reports9040340)
Supplement: Supplementary file 1 [file reports-09-00340-s001.zip › reports-4577266-supplementary.pdf]

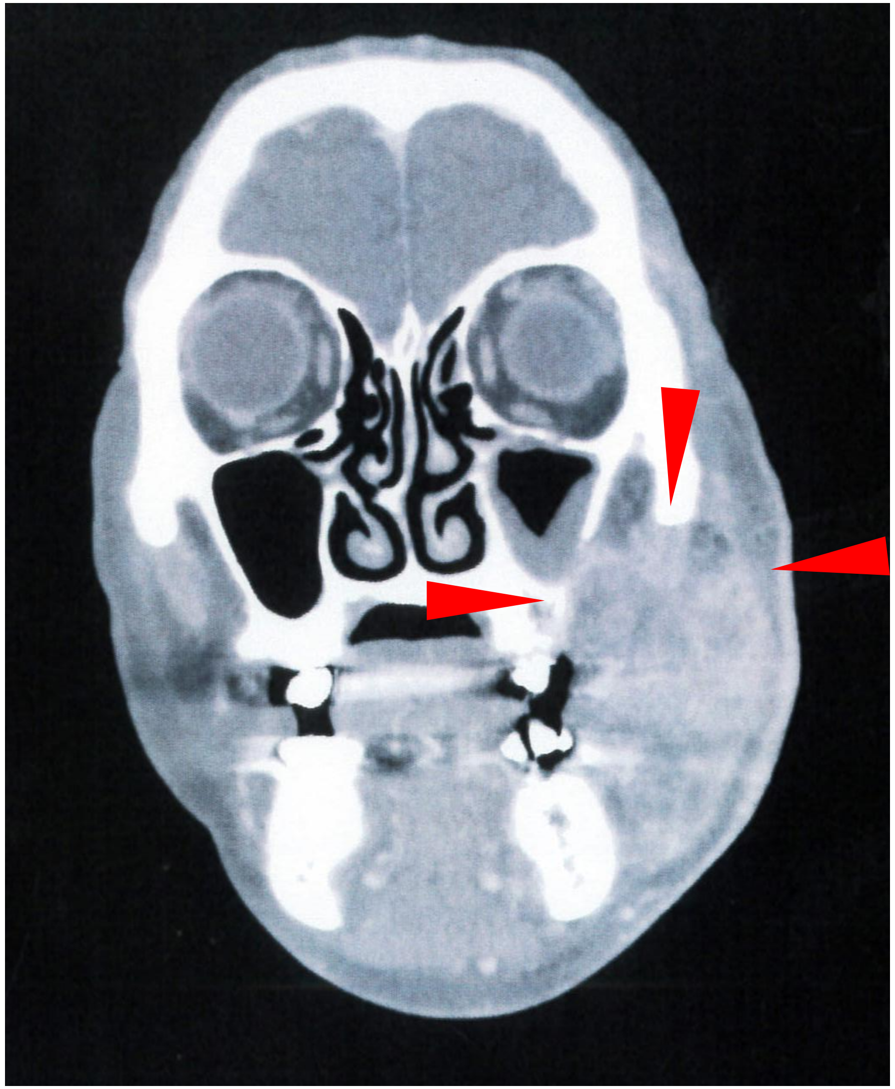

Supplementary Figure S1

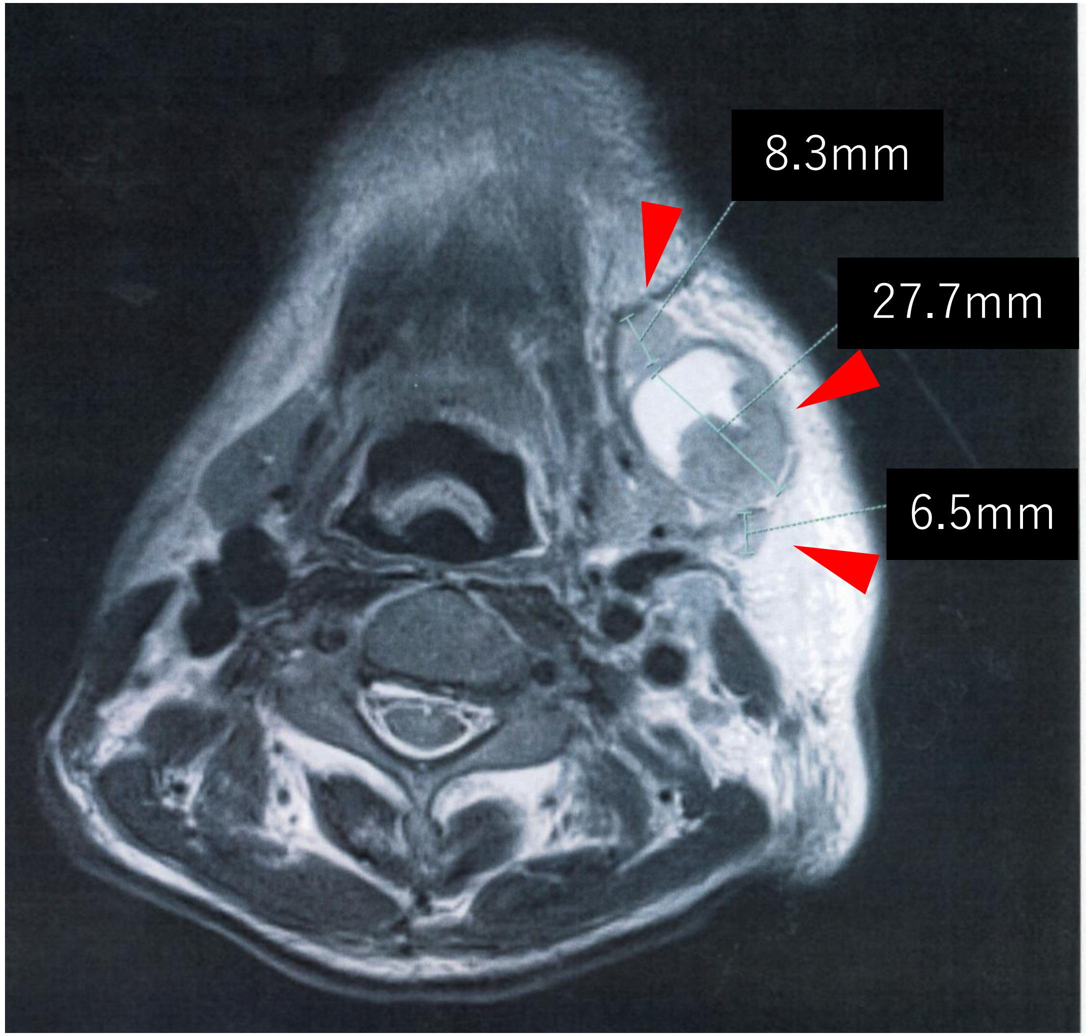

Supplementary Figure S2

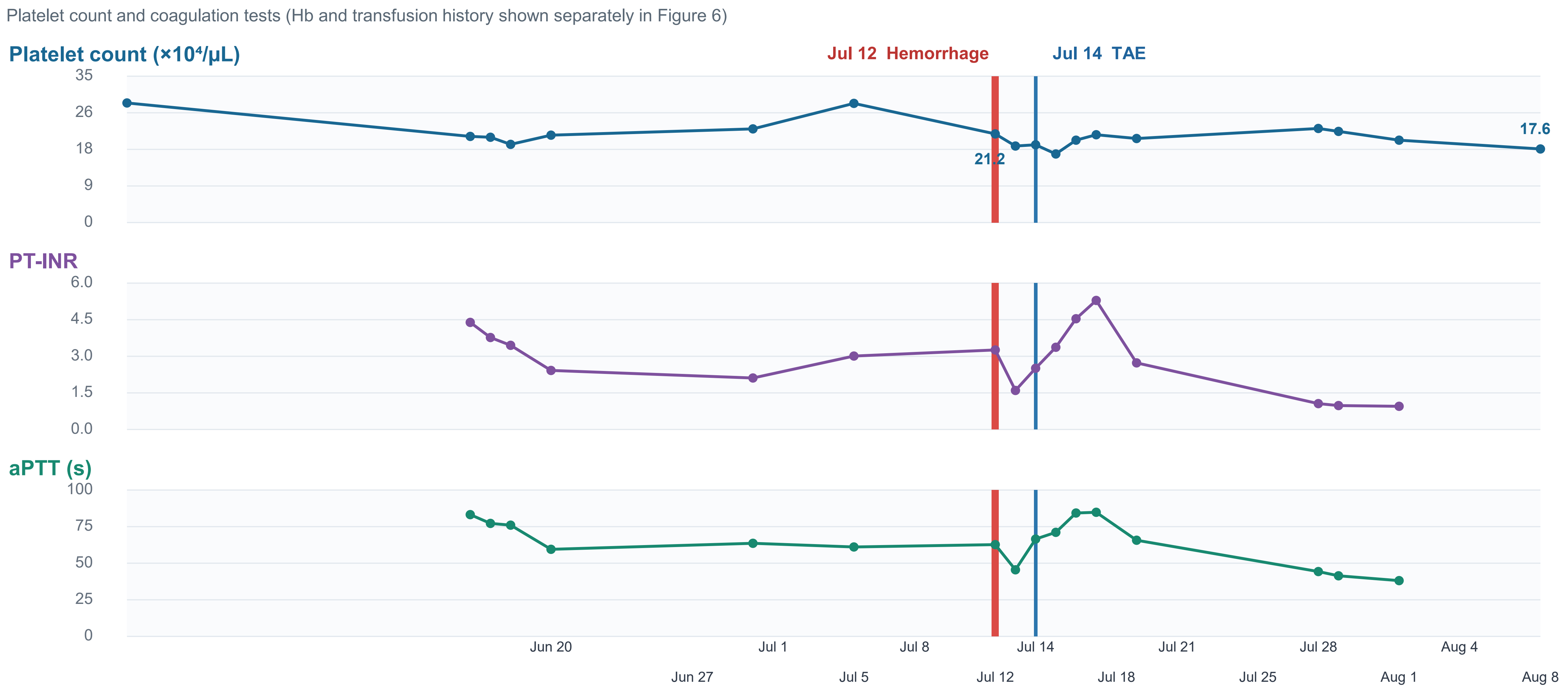

Institutional reference ranges: PT-INR, 0.85–1.15; aPTT, 20.0–40.0 s.

Supplementary Figure S3 | Serial laboratory parameters before and after tumor hemorrhage
